# Psychometric Properties of the Bern Illegitimate Tasks Scale – Spanish Version

**DOI:** 10.3389/fpsyg.2021.593870

**Published:** 2021-03-17

**Authors:** Denisse Lizette Valdivieso Portilla, Angélica Gonzalez Rosero, Geovanny Alvarado-Villa, Jorge Moncayo-Rizzo

**Affiliations:** ^1^Occupational Safety and Health Program, Universidad de Especialidades Espíritu Santo, Samborondón, Ecuador; ^2^Ecuadorian Social Security Institute (IESS), Quito, Ecuador; ^3^Medicine School, Universidad de Especialidades Espíritu Santo, Samborondón, Ecuador

**Keywords:** Bern Illegitimate Tasks Scale, illegitimate tasks, psychometric properties, confirmatory factor analysis, exploratory factor analysis, cross-cultural validation

## Abstract

In recent years, a new factor for work stress has been studied along with stress as an offense to self-theory. Illegitimate tasks refer to assignments that are unnecessary or are not related to the employee’s role. Because of this, the Bern Illegitimate Tasks Scale was developed, which measures illegitimate tasks in terms of unreasonable tasks and unnecessary tasks. There are no studies in Latin America on illegitimate tasks, so the purpose of this research is to translate and validate the Bern Illegitimate Tasks Scale. The study was performed with a sample of nursing staff from a hospital in Guayaquil, Ecuador. Written informed consent was obtained from each of the participants. The reliability of the questionnaire was evaluated and its structural validity was verified by exploratory factor analysis and confirmatory factor analysis. The internal consistency of the whole scale, measured by Cronbach’s alpha, was 0.857. Moreover, the unnecessary and unreasonable subscales measure were 0.846 and 0.841, respectively. The exploratory factor analysis supported a two-factor model that explained 73.96% of the variance. Additionally, the confirmatory factor analysis showed good indexes of fit (GFI = 0.915, CFI = 0.955, TLI = 0.933, SRMR = 0.084, and RMSEA = 0.087). The Spanish version of the Bern Illegitimate Tasks Scale presents good psychometric properties and can be applied to nurses in the Ecuadorian population.

## Introduction

The assignment of tasks in the workplace is a critical step that can directly influence the emotional and psychosocial environment of employees. This is described in the Stress as Offense to Self-theory (SOS), which considers that assigning tasks that are not within the expectation of the employee will be regarded as an offense to the self (Semmer et al., 2007, 2010). SOS theory analyses two aspects of the individual (Semmer et al., 2019). The first aspect is the personal self, which refers to the moral aspects of one’s work, behavior and performance in the workplace (Semmer et al., 2007, 2019). The second aspect refers to the social self, which analyses the impact of social value, that is, one’s perception of feeling accepted or not by others (Semmer et al., 2007, 2019; Pfister et al., 2020a). When these two aspects promotes negative emotions in the individual, causes an individual to perceive threats to the self, or affects personal self-esteem, they are defined as stress-as-insufficiency (SIN) and stress-as-disrespect (SAD), respectively (Semmer et al., 2007, 2019).

In addition, SOS theory not only describes threats to self-esteem but also assesses boosts to self-esteem (Semmer et al., 2007; Pfister et al., 2020b), which imply success and failure, meaning that workers who achieve goals or make progress toward goals are less able to develop SIN (Semmer et al., 2007, 2019). Similar to SIN, SAD can be lowered by positive affect and satisfaction (Semmer et al., 2019, 2020; Pfister et al., 2020a,b; Stein et al., 2020). Therefore, these two factors are the principal states of SOS theory.

However, SOS theory also indicates that stressors can threaten the social identity of workers, sometimes referring to them as identity stressors (Semmer et al., 2010, 2020; Ma and Peng, 2019). This means that an employee will perceive a task that may be outside of the range of his/her occupation as offensive as a consequence of the social identity that is formed by the professional role of the employee (Semmer et al., 2010; Björk et al., 2013; Ma and Peng, 2019). This phenomenon represents a factor for job stress that can result in counterproductive behavior and has therefore been assumed to be associated with mental health (Semmer et al., 2010; Eatough et al., 2016; Munir et al., 2017). This phenomenon also depends on the emotional status of employees (Semmer et al., 2007, 2019).

Much of the time job exigencies and pressure are mistaken for work challenges that keep employees motivated. Therefore, an inadequate assignment of tasks can saturate employees with excessive demand and pressure, leading to work stress (Semmer et al., 2019). Tasks that create an offense to the self are called “illegitimate tasks”(Semmer et al., 2007, 2010, 2019). These illegitimate tasks can be classified as unreasonable or unnecessary. Unreasonable tasks are those that are not part of the role of employees, whereas unnecessary tasks are those tasks that can be carried out by another person or are simply expendable within a process (Semmer et al., 2010, 2019).

However, it has been observed that the legitimacy of tasks depends on the context in which the tasks are developed (Semmer et al., 2015), which means that there is an important factor that involves the subjective perception of the worker. That is, the employee would not consider a task illegitimate if he/she accepts it or if it is part of his/her initiative (Semmer et al., 2015).

Although SOS theory and illegitimate tasks apply to every type of work, health care professionals (HCPs) are generally exposed to a high workload and multiple stressors, leading to high rates of absenteeism (Thun et al., 2018), burnout, and diseases (Brand et al., 2017; Anskär et al., 2019). Nurses represent the largest group of healthcare professionals and provide patient care 24 h a day. Nursing personnel are exposed to work situations in which there is a lack of autonomy for decision-making, a high workload and a lack of professional recognition (Öksüz et al., 2019). In addition, there is ambiguity in the definition of their role, which is referred to as illegitimate tasks, resulting in chronic stress and a low level of job satisfaction (Lu et al., 2012; Anskär et al., 2019).

To assess illegitimate tasks, the Bern Illegitimate Tasks Scale was developed by Jacobshagen (2006). This questionnaire can assess both unreasonable and unnecessary tasks with four questions each. Since illegitimate tasks have been suggested to be a stressor, several studies have investigated their association with other variables and conditions.

Kottwitz et al. (2013) suggests that illegitimate tasks predict cortisol levels in participants who rated their health comparatively low (*b* = 1.43, *p* < 0.01). Verkuilen et al. (2020) report that unreasonable tasks and unnecessary tasks are positively correlated with burnout (*r* = 0.4 and 0.36) and depression (*r* = 0.42 and 0.37) and are predictors of general distress. Pereira finds that illegitimate tasks are negatively related to sleep quality; specifically, they are positively related to sleep fragmentation and sleep latency (Pereira et al., 2014). Illegitimate tasks are also negatively correlated with job satisfaction (Omansky et al., 2016; Kottwitz et al., 2019; Ilyas et al., 2021). Moreover, it has been shown that illegitimate tasks are correlated with work-to-family conflict (Jacobshagen, 2006), and Zhou et al. (2020) reports an indirect effect of illegitimate tasks on work-to-family conflict via psychological detachment. Finally, illegitimate tasks are a negative predictor of work well-being along with other stressors, such as a lack of social support, work overload, high demands, and low resources (Hirschle and Gondim, 2020).

Even now, due to the COVID-19 pandemic, managers’ mental health has been impaired. A study carried out by Graf-Vlachy determines the predictors of managers’ mental health. The results show that illegitimate tasks predict distress, anxiety and depression (Graf-Vlachy et al., 2020).

Moreover, illegitimate tasks have been studied in HCPs. Reports from Sweden (Anskär et al., 2019), Norway (Thun et al., 2018) and Germany (Stein et al., 2020) show that HCPs are exposed to high levels of illegitimate tasks. However, to our knowledge, investigations involving illegitimate tasks in nursing personnel have not taken place in any Latin American country. Therefore, the main purpose of this study is to translate and validate the Bern Illegitimate Task Scale (BITS) for its use in Ecuador for future research on job stressors.

## Methodology

### Sample

The present study was performed using a sample of nursing personnel at the Teodoro Maldonado Carbo Hospital in Guayaquil city. Nurses who have administrative roles or antecedents of psychiatric diseases were excluded. According to Suhr (2006) and similar validation studies performed in Ecuador (Alvarado-Villa et al., 2019), the sample size should be in the range of 40–160 participants. Additionally, when calculating the sample size, considering that 20% of responses contained missing values, the sample required for the present study was 136 participants. Informed consent was obtained from each patient who wanted to participate in the study.

### Translation

The method for translating the instrument was performed according to Sperber (Sperber, 2004). The method consisted of two phases: the first phase was reverse translation and the second phase was the interpretability and comparability of the instrument. The translation into Spanish was performed by two native speakers from Ecuador with a fluent command of the English language. Both translations were compared, and a single version was developed for each question. Then, the translation back into English was performed by a certified translator. Neither of the translators knew the concept nor the purpose of the instrument.

For the second phase, the questionnaire was administered to 30 people with a fluent command of both languages. Each question was qualified using two criteria: A – comparability of language and B – similarity of interpretability. The scoring was performed using a Likert scale: 1 (extremely comparable/extremely similar), 4 (moderately comparable/moderately similar) and 7 (nothing comparable/nothing similar). The score was considered acceptable if the comparability was below 3 and the interpretability was below 2.5. If the items did not satisfy the score, then they were re-evaluated.

### Data Collection

Data collection was carried out in a scheduled visit. The data collected included sociodemographic and organizational characteristics (type of contract, length of employment, shift, etc.) and the BITS. The questionnaire was applied electronically on the QuestionPro platform. However, a physical form was used as a guide if the participant required it or the researcher determined the need.

### Statistical Analysis

Qualitative variables are presented as frequencies using percentages, while quantitative data are represented with means and standard deviations. The reliability of the questionnaire was assessed by Cronbach’s alpha, which had a minimum acceptable value of 0.7. To perform the factor analysis, the sample was randomly split into two halves using the statistical program SPSS version 23 for Windows. The decision to perform an exploratory factor analysis was made because the original version was assessed with principal component analysis (PCA) (Jacobshagen, 2006), which was not appropriate. This is because PCA implies that the observed variables are uncorrelated and that no unobserved variables are underlying the observed variables (Joliffe and Morgan, 1992; Suhr, 2005). Moreover, the decision to use EFA was supported because there was no previously validated Spanish version of the BITS, and there is evidence from other validations that dimension reduction analysis is needed (Salanova and Schaufeli, 2000; Alvarado-Villa et al., 2019; Cherrez-Ojeda et al., 2019; Bomfim et al., 2020).

The first half was used to perform exploratory factor analysis. The Kaiser-Meyer-Olkin (KMO) test and Bartlett’s sphericity test were performed to test the adequacy of the sample. The KMO test result of >0.7 and Bartlett’s sphericity test result of *p* < 0.05 indicated that the sample was adequate for factor analysis. Maximum likelihood was used to extract factors and was confirmed with scree plots and Veciler’s minimum average partial (MAP) test. Finally, the rotation used to determine the items of each factor was direct oblimin.

Confirmatory factor analysis was performed in the second half of the sample to demonstrate the validity of the factorial structure of the construct. The indexes used were the following: the comparative adjustment index (CFI), the goodness of fit index (GFI), the Tucker Lewis Index (TLI), the root mean square error of approximation (RMSEA), and the standardized root mean square residual (SRMR). According to Hu and Bentler (1999), the indexes should be CFI, GFI, and TLI >0.9 and RMSEA and SRMR <0.08. Additionally, intercorrelation of errors would be permitted if necessary, and the theory justified it. Confirmatory factor analysis was performed with the AMOS application from SPSS.

## Results

This study included the participation of 142 members of the HTMC nursing area. Table 1 shows the variables obtained from the participants. Of the 142 participants, only 11 presented missing values in the “age” variable. Therefore, among the 131 participants, the mean age was 33.2 years (SD: ±8.84). The response rate for the BITS was 100%. For the whole sample, 73.2% of the participant were females, more than half had children (57%) and 42.96% were single. Regarding organizational characteristics, 50% of the participant had an occasional type of contract, 19.72% had more than 5 years of work experience, almost half worked a morning shift (47.89%); and 35.9% performed overtime hours. Regarding the item scores, the mean ranged from 2.35 to 3.14. The skewness ranged from −0.124 to 0.455, and the kurtosis ranged from −0.409 to −0.954. The descriptive statistics of the items are shown in Table 2.

**TABLE 1:** Demographic and organizational characteristics.

Variables		Count (*N* = 142)	Percentage
Sex	Female	104	73.24
	Male	38	26.76
Marital status	Married	53	37.32
	Single	61	42.96
	Divorced	12	8.45
	Widowed	4	2.82
	Civil union	12	8.45
Children	Yes	81	57.04
	No	61	42.96
Type of contract	Occasional	71	50.00
	Provisional	35	24.65
	Permanent	32	22.54
	Other	4	2.82
Length of employment (years)	1–2	71	50.00
	3–5	43	30.28
	>5	28	19.72
Working day	Morning	68	47.89
	Afternoon	11	7.75
	Evening	10	7.04
	Rotating	53	37.32
Overtime hours	Yes	51	35.92
	No	91	64.08

**TABLE 2:** Descriptive statistics of the items of the BITS.

		Count	% de *N*	Mean (SD)	Kurtosis	Skewness
¿Usted tiene tareas de trabajo por hacer, que lo mantienen preguntándose si…						
*Do you have work tasks to take care of, which keep you wondering if…*						

1…tienen que hacerse completamente?	Never	27	19.0	2.76 (1.31)	0.358	−0.899
	Almost never	39	27.5			
	Sometimes	39	27.5			
*…they have to be done at all?*	Frequently	15	10.6			
	Very frequently	22	15.5			

2…tienen sentido?	Never	20	14.1	3.03 (1.25)	−0.032	−0.931
	Almost never	28	19.7			
	Sometimes	43	30.3			
*…they make sense at all?*						
	Frequently	30	21.1			
	Very frequently	21	14.8			

3…no existirían (o podrían hacerse con menos esfuerzo), si se organizaran de manera diferente?	Never	19	13.4	3.14 (1.28)	−0.124	−0.954
	Almost never	24	16.9			
	Sometimes	43	30.3			
*…they would not exist (or could be done with less effort), if it were organized differently?*						
	Frequently	30	21.1			
	Very frequently	26	18.3			

4*…* solo existen porque algunas personas simplemente lo exigen de esta manera?	Never	27	19.0	2.77 (1.16)	−0.043	−0.789
	Almost never	25	17.6			
*…they just exist because some people*	Sometimes	52	36.6			
	Frequently	29	20.4			
*simply demand it this way?*						
	Very frequently	9	6.3			

¿Tiene tareas de trabajo que hacer, que cree que…						
*Do you have work tasks to take care of, which you believe…*						

5*…* deben ser hechas por otra persona?	Never	44	31.0	2.35 (1.17)	0.455	−0.663
	Almost never	34	23.9			
	Sometimes	41	28.9			
*…should be done by someone else?*						
	Frequently	16	11.3			
	Very frequently	7	4.9			

6*…* van demasiado lejos, algo que no debería esperarse de usted?	Never	36	25.4	2.44 (1.16)	0.431	−0.582
	Almost never	40	28.2			
	Sometimes	41	28.9			
*…are going too far, which should not be expected from you?*						
	Frequently	17	12.0			
	Very frequently	8	5.6			

7*…* lo ponen a usted en una posición incómoda?	Never	34	23.9	2.43 (1.11)	0.419	−0.409
	Almost never	41	28.9			
	Sometimes	46	32.4			
*…put you into an awkward position?*						
	Frequently	14	9.9			
	Very frequently	7	4.9			

8…es injusto que usted tenga que lidiar con ellas?	Never	34	23.9	2.54 (1.21)	0.433	−0.562
	Almost never	36	25.4			
*…are unfair that you have to deal with them?*	Sometimes	46	32.4			
	Frequently	13	9.2			
	Very frequently	13	9.2			

The reliability of the questionnaire, calculated by Cronbach’s alpha, was 0.857. Each of the items of the scale had a corrected item-total correlation of more than 0.4. Moreover, the reliability for each subscale was 0.846 for unnecessary tasks and 0.841 for unreasonable tasks (see Table 3).

**TABLE 3:** Factor loadings, corrected item-total correlation, and Cronbach’s alpha.

Items	Factor		Corrected item-total correlation	Cronbach’s alpha
	1	2		
BITS Question 1		−0.755	0.551	0.846
BITS Question 2		−0.865	0.540	
BITS Question 3		−0.881	0.662	
BITS Question 4		−0.744	0.697	
BITS Question 5	0.537		0.477	0.841
BITS Question 6	0.840		0.560	
BITS Question 7	0.789		0.687	
BITS Question 8	0.896		0.641	

*Extraction method: maximum likelihood. Rotation method: oblimin with Kaiser normalization.*

### Exploratory Factor Analysis

As described previously, for the factor analysis (both exploratory and confirmatory), the sample was randomly split into two halves. In the first sample, Bartlett’s sphericity test (*x*^2^ 235.79; df = 28; *p* < 0.001) and the KMO test (0.831) were performed, which showed that exploratory factor analysis was appropriate. With this analysis, two factors were determined that explained 73.96% of the variance. The scree-plot test and Veciler’s MAP test supported the decision to retain two factors. The first factor (which explained 51.86% of the variance) corresponded to items 5–8; meanwhile, the second factor (which explained 22.1% of the variance) corresponded to items 1–4. All the items had a factor loading greater than 0.5 in their respective factor (see Table 3). The highest loading for factor one was item 8 (0.896) and for factor two was item 3 (−0.881). No cross-loadings were present, except for item 4 which had a loading on factor 1 (0.633). The factors were negatively correlated (*r* = −0.376) according to the oblimin rotation.

### Confirmatory Factor Analysis

**FIGURE 1:**
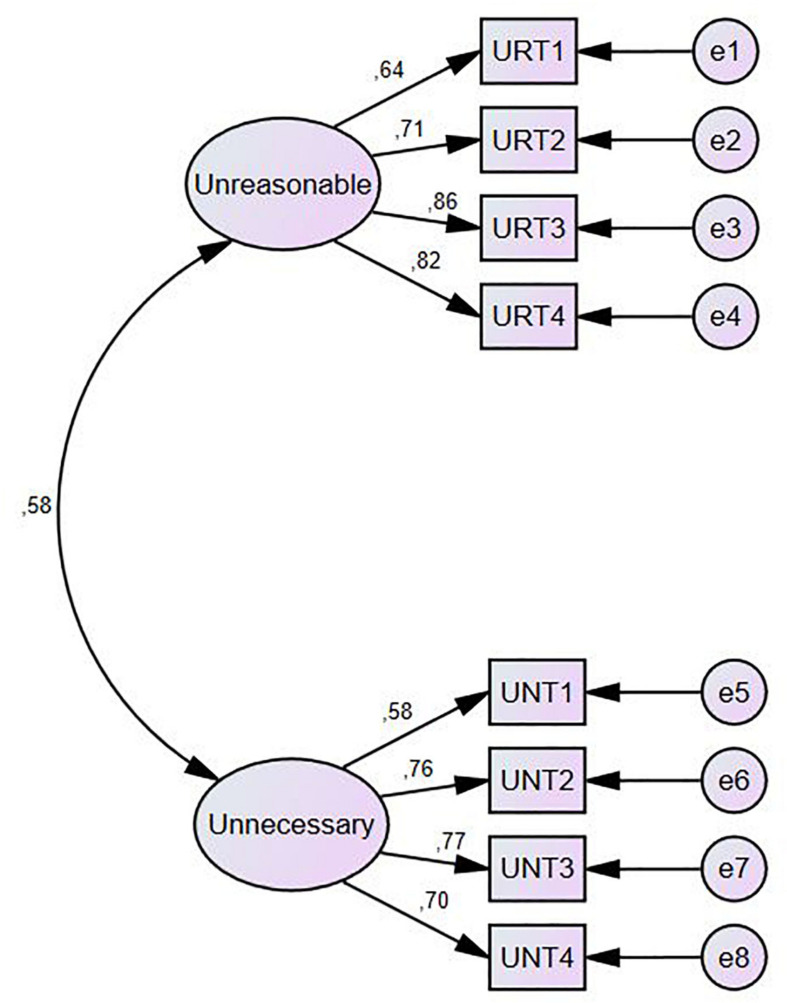
Model 1 for illegitimate tasks’ construct.

Confirmatory factor analysis was performed in the second half of the sample. The model used was determined by exploratory factor analysis (Figure 1). *x*^2^ 28.982 and *p* = 0.066 indicated the homogenous distribution of the data over the model. Finally, the indexes of the fit were (see Table 4): GFI = 0.915, CFI = 0.955, TLI = 0.933, SRMR = 0.084, and RMSEA (CI-90%) = 0.087 (0.000 – 0.147), indicating a moderate to good quality-of-fit.

**TABLE 4:** Confirmatory factor analysis.

Model	X^2^	Degree of freedom	*p*-value	GFI	CFI	RMSEA (CI-90%)	TLI	SRMR
Model 1	28.982	19	0.066	0.915	0.955	0.087 (0.000 – 0.147)	0.933	0.084
Jacobshagen’s Model	312.30	19	0.000	0.95	0.83	0.07 (0.07 – 0.08)	0.750	0.050

## Discussion

This study presents the psychometric properties of the Latin American Spanish version of the BITS developed by Jacobshagen (2006). The translation process was performed according to Sperber (2004). Analysis of the results was performed in contrast to the outcomes of Jacobshagen.

The Spanish version of the BITS showed good internal consistency for both the whole scale (Cronbach’s alpha = 0.857) and the subscales (see Table 3). Moreover, many studies have demonstrated good reliability for the whole scale (Cronbach’s alpha = 0.79–0.88), the unnecessary task subscale (Cronbach’s alpha = 0.76–0.91), and the unreasonable task subscale (Cronbach’s alpha = 0.73–0.91) (Jacobshagen, 2006; Semmer et al., 2010; Pereira et al., 2014; Muntz et al., 2019; Pfister et al., 2020b).

The exploratory factor analysis, using the extraction method of maximum likelihood with the rotation method of direct oblimin, established two factors that explained 73.96% of the variance. In contrast to that presented by Jacobshagen, these factors explained 63.61% of the variance (Jacobshagen, 2006). In addition, it is important to mention that the method used to extract factors by Jaboshagen was PCA. PCA is not considered an appropriate method for factor analysis because of the assumptions that it involves, in contrast to EFA, which hypothesizes the underlying construct (Suhr, 2005; Howard, 2016).

Finally, the confirmatory factor analysis showed similar results compared to those presented by Jacobshagen (2006) (see Table 4), except for RMSEA and SRMR, which were over the cut-off score. The indexes presented in our study indicated that the model fit adequately to the sample. Although we had a small sample for the confirmatory analysis, the results were supported by the theoretical fundaments developed by Jacobshagen (2006) and by the exploratory factor analysis performed by us, for which the sample size was appropriated (Suhr, 2006).

The limitations of this study include the small sample size, so the results cannot be generalized to other Spanish-speaking countries. Additionally, the sample only included nursing staff, so CFA should be performed when applied to other professions. The strengths of the study are that the correct methodological process was performed by using EFA instead of PCA and that this is the first translation of the BITS into Spanish. Moreover, as stated by Squires et al. (2013), an instrument can have a good translation, but the relevance of the questions may not score well in a different context if they are not analyzed. Finally, this allows future research to investigate illegitimate tasks as a stressor in Latin America.

In conclusion, illegitimate tasks are a topic that is gaining interest in scientific areas such as occupational medicine, psychology and psychiatry. Illegitimate tasks have been demonstrated to be an important factor in work-stress production and counterproductive behavior development. Due to this, the BITS has been developed, which allows for the measurement of the effects of illegitimate tasks. Now, with this study, the questionnaire can be applied to nursing staff in Ecuador.

## Data Availability Statement

The raw data supporting the conclusions of this article will be made available by the authors, without undue reservation.

## Ethics Statement

The studies involving human participants were reviewed and approved by the Comité de Ética del Hospital Clínica Kennedy. The patients/participants provided their written informed consent to participate in this study.

## Author Contributions

DV and AG were responsible for the data collection and literature. GA-V and JM-R performed the statistical analysis. DV, GA-V, and JM-R contributed to the edition of the manuscript. All authors contributed to the article and approved the submitted version.

## Conflict of Interest

The authors declare that the research was conducted in the absence of any commercial or financial relationships that could be construed as a potential conflict of interest.
